# Lessons Learned from Japan’s Response to the First Wave of COVID-19: A Content Analysis

**DOI:** 10.3390/healthcare8040426

**Published:** 2020-10-23

**Authors:** Kazuki Shimizu, Masashi Negita

**Affiliations:** 1 Department of Health Policy, London School of Economics and Political Science, Cowdray House, Houghton Street, London WC2A 2AE, UK; 2 Faculty of Public Health and Policy, London School of Hygiene and Tropical Medicine, Keppel Street, London WC1E 7HT, UK; 3 Graduate School of Medicine, Hokkaido University, Kita 15 Nishi 7, Kita-ku, Sapporo 060-8638, Japan; 4 Department of Surgery, Komaki City Hospital, 1-20, Jobushi, Aichi, Komaki 485-8520, Japan; mnegita-ngy@umin.ac.jp

**Keywords:** COVID-19, coronavirus, infectious disease epidemiology, health system, testing, health communication, crisis communication, leadership, governance, Japan

## Abstract

While the epidemiological impact of the coronavirus disease 2019 (COVID-19) pandemic has been relatively moderate in East-Asian countries, the pandemic has significantly impacted on citizens’ lives and livelihoods, and Japan is no exception. In the early phase of the COVID-19 pandemic, Japan managed unprecedented quarantines and realized the difficulty of controlling COVID-19, finally recording a relatively high number of deaths per million in the Western Pacific region. However, scant research has highlighted the distinctive features of Japan’s reaction and the challenges encountered. To clarify these points and examine Japan’s first response to COVID-19, we performed a content analysis. Minutes of expert meetings were analyzed from multiple viewpoints, including epidemiology, health systems, border control, and health communication. The obscure evolution of the testing strategy, the usefulness of retrospective contact tracing, the rapid scientific risk assessment, a sluggish expansion of health system capacity and response in border control, and misunderstanding between risk communication and crisis communication are made evident by our analysis. Examining previous responses and gathering lessons learned in each country will improve global responses to COVID-19 and strengthen regional health security. Therefore, while investing in public health and ensuring transparency, Japan needs to clarify the previous decision-making process of each countermeasure towards COVID-19.

## 1. Introduction

While the coronavirus disease 2019 (COVID-19) pandemic has significantly influenced citizen’s lives and livelihoods, the epidemiological impact of COVID-19 has been relatively moderate in the Western Pacific region [1]. East-Asian countries’ rapid preparedness and response to the pandemic have resulted in a lower number of cases and deaths per population compared to those recorded in Western countries [1]. The COVID-19 response in Taiwan, represented by a fully digitalized response with an early and decisive government action, appears to be a good model [2,3,4]. In South Korea, the government responded swiftly by thoroughly implementing principles of pandemic response: massive testing, contact tracing, case isolation, and quarantine [5,6,7]. The emergent expansion of health system capacity, the effectiveness of lockdown, and massive testing for all citizens in containment phases in China have offered a great number of scientific insights [8,9,10,11].

Japan, a neighboring country of China, reported the first case of COVID-19 in mid-January and subsequently experienced unprecedented quarantines in anticipation of a pandemic situation. From late February, a cluster-based approach was employed [12,13], followed by a state of emergency in April–May. Japan could respond to the first wave of COVID-19 without a strict lockdown, and Prime Minister (PM) Abe proudly described his country’s response as the “Japan Model” [14], but the specifics of how Japan was able to curb the epidemic have not been explored persuasively. Despite the relatively lower number of infections, the loss of Gross Domestic Product (GDP) in April–June was evident compared to other East-Asian countries [15], and Japan’s handling of COVID-19 underwent a relatively limited evaluation [16]. Furthermore, the number of COVID-19 deaths per million people in Japan is higher than in other countries of the Western pacific region [1].

Reflecting and acting upon lessons learned during the pandemic can help us to build and revise strategies of containment and thus mitigate the impact of COVID-19 and other pandemic scenarios in the future [12,17]. However, when lifting the state of emergency in late May, PM Abe claimed that, as a nation, Japan “is still not being investigated” and “verification will be conducted after the end of COVID-19” [18]. Both distinctions and challenges in Japan’s response to the first wave of COVID-19 have not been internally and externally discussed enough, except in a few articles [12,19,20,21].

## 2. Materials and Methods

The primary objective of this case study was to clarify how Japan responded to the first wave of the COVID-19 pandemic. We undertook three steps during our investigation. First, we identified and recorded epidemiological trends of COVID-19 in Japan in January–May, 2020, through the official website of the Ministry of Health, Labor, and Welfare, Japan [22], and reviewed policy measures. Second, we identified 12 expert meetings, including 2 advisory board meetings, that were convened before the declaration of the state of emergency on 7 April 2020. As transcripts of the meetings have been withheld from the public, we collected publicly available minutes of advisory board meetings and expert meetings [23,24,25,26,27,28,29,30,31,32,33,34] and “views” or “analysis and recommendations” related to the COVID-19 response [35,36,37,38,39,40,41,42,43] and conducted a content analysis, as performed elsewhere [5,6,44]. Each minute was specifically analyzed by thematic factors that contributed to the declaration of the state of emergency. These included: epidemiological investigation (i.e., testing and tracing), health system capacity, border control, and health communication. These meetings were occasionally followed by a press conference, in which the chair, vice-chair, and some participants explained the main discussion points and answered inquiries from the press; the results of these debates were excluded from the analysis. As this study analyzed secondary datasets that were anonymized in advance and made publicly available, patients and the public were not involved in this study, and ethical approval by an institutional review board was not required.

## 3. Epidemiological Trends of COVID-19 and Policy Measures in Japan, January–May 2020

**Figure 1:**
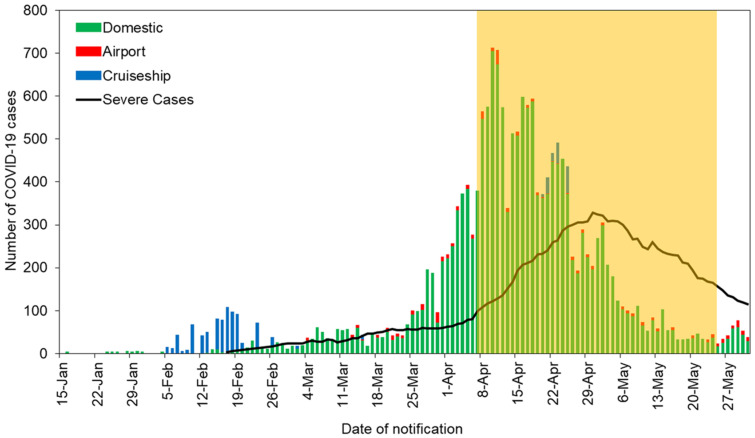
Number of coronavirus disease 2019 (COVID-19) cases in Japan, January–May, 2020, by date of notification.

This section outlines the timeline and policy measures related to COVID-19 in Japan. As of the end of May, 16,884 cases with 892 deaths were reported, in addition to 860 cases in the cruise ships Diamond Princess and Costa Atlantica [22]. The evolution of COVID-19 in Japan is presented in Figure 1.

Domestic cases are shown in green, and cases detected in airport quarantine are shown in red. Cases detected in cruise ships are presented in blue. The black line indicates the evolution of severe COVID-19 cases, as presented by the Ministry of Health, Labor, and Welfare, Japan. The yellow shadow highlights the period of Japan’s state of emergency (declared in at least one prefecture) in Japan.

We divided Japan’s response in January–May into four periods: (i) early January to 25 February 2020; (ii) 25 February 2020 to 7 April 2020; (iii) 7 April 2020 to 25 May 2020; and (iv) after 25 May 2020. In the first period, Japan hastily launched a command and control approach while experiencing unprecedented quarantines. Thereafter, Japan started to employ a cluster-based approach, which was followed by the declaration of the state of emergency on 7 April 2020. This was lifted in all prefectures on 25 May 2020. Key policy measures are summarized in Table 1.

### 3.1. Early January to 25 February 2020: Lauching a Command and Control Approach

On 16 January 2020, the first COVID-19 case who had a travel history to Wuhan was reported in Japan [22], and subsequently, other COVID-19 cases without travel history to Wuhan within 14 days were reported on 28 January 2020 [22]. COVID-19 response headquarters were launched on 30 January 2020 [45], and thereafter, Japan managed two quarantines related to COVID-19: a Wuhan repatriation mission (evacuation flights from Wuhan) and a quarantine on the Diamond Princess. Between 29 January 2020 and 17 February 2020, five chartered flights returned to Japan to repatriate citizens from Wuhan [46]. Moreover, the quarantine of the cruise ship Diamond Princess, that started on 3 February 2020, was critically controlled [47,48].

Consultation criteria for COVID-19 testing were published by the Ministry of Health, Labor, and Welfare on 17 February 2020. They instructed that, with the exception of the elderly, patients with previous medical histories, pregnant women, and people who had symptoms of cold or fever above 37.5 °C for four consecutive days needed to consult the Coronavirus Consultation Center [49].

### 3.2. 25 February 2020 to 7 April 2020: Cluster-Based Approach and Proactive Actions at Local Levels

On 25 February 2020, the fundamental plan of action “Basic Policies for Novel Coronavirus Disease Control” was introduced [50], and on the same day, the cluster-response section, composed of epidemiologists and data analysts, was launched within the Ministry of Health, Labor, and Welfare. In Hokkaido, school closure was requested on 26 February 2020, and a local state of emergency was declared on 28 February 2020 [51]. A controversial request for nationwide school closure from 2 March 2020 to the end of spring vacation was announced on 27 February 2020, which was subsequentially cancelled on 20 March 2020 [52]. Postponement of the Tokyo 2020 Olympics and Paralympics was announced on 24 March 2020 [53], which was followed by a press conference of Tokyo governor on 25 March 2020 that expressed critical concerns on an explosive increase of COVID-19 cases [54]. Afterwards, the necessity of decreasing social contacts by 80% was announced by an epidemiologist of the cluster-response section [55].

Border control became an important concern for the Japanese government during this period. Under the Immigration Control, foreign nationals who had stayed in any of the specified countries or regions within 14 days, or held a passport issued in Hubei or Zhejiang provinces in China, or had boarded one of the quarantined cruise ships (Westerdam) that had departed from Hong Kong were denied entry to Japan, unless there were exceptional special circumstances in mid-February [56,57]. Airport quarantine was gradually strengthened for visitors from specific areas, and people who had a travel history to “areas specified as in strengthened quarantine” or “epidemic areas” designated by immigration restrictions within 14 days, were requested to be quarantined for 14 days at locations appointed by the chiefs of quarantine stations and under no circumstances to use public transportation networks [56,57]. Furthermore, all passengers who visited designated countries and areas were screened by polymerase chain reaction (PCR) testing and followed by occasional health checks by public health centers [56,57]. The evolution of the safety measures in designated areas and countries are listed in Table 2.

### 3.3. 7 April 2020 to 25 May 2020: The State of Emergency

PM Abe declared the state of emergency in seven prefectures on 7 April 2020, which was expanded to the entire nation on 16 April 2020 [67]. Japan’s state of emergency was different from the “lockdown” with enforceability applied in many countries. While governors at prefectural levels could ask citizens to refrain from getting out, the state of emergency was dependent on citizens’ modification of their behavior on a voluntary basis [68]. PM Abe argued in the press conference on 7 April 2020 that decreasing social contacts by “at least 70% and ideally 80%” was vital to curb the epidemic in two weeks [69]. However, he weakened his argument by noting that this was not a “lockdown like in other countries” and ensured that services such as public transportations would be maintained [69]. On 4 May, the state of emergency period was expanded until the end of May [70]. The government presented examples of the demanded “new lifestyle” and requested citizens to adopt it during the COVID-19 pandemic.

The extended state of emergency was lifted in 39 prefectures on 14 May 2020 by “comprehensive judgement [71]”. On 21 May 2020, it was also lifted in additional three prefectures in Kansai region [72]. Although the new infections per 100,000 people were still above 0.5 per 100,000 population in Kanagawa and Hokkaido prefectures, the state of emergency was finally lifted in all prefectures on 25 May 2020 [73].

### 3.4. After 25 May 2020

Some clusters were reported during this period. In particular, in Kitakyushu city, in which no new COVID-19 cases had been recorded between 30 April 2020 and 22 May 2020, 97 cases were reported between 23 May 2020 and 31 May 2020 [74]. Due to active infections, public facilities were closed again.

As the data of COVID-19 aggregated by age group were weekly updated by the Ministry of Health, Labor, and Welfare, the number of confirmed cases and deaths as of 27 May 2020—the closest date to the lift of the state of emergency on 25 May 2020—was extracted and is presented in Table 3.

## 4. Results

There were 2 advisory board meetings and 10 expert meetings before the declaration of the state of emergency. They were launched to advice the government from a medical perspective and support the decision-makers during the COVID-19 pandemic. The expert meeting was composed of 12 members, and 9 were licensed physicians. Their specialties were mainly virology, infectious disease epidemiology, public health, and clinical infectious diseases. One lawyer and one professor in medical sociology were also included; however, experts in key disciplines, such as behavioral sciences, media and communication, and economics were not included. To compensate for this drawback, a chairman could additionally ask other experts to attend the meeting. While transcripts have not been open to the public, minutes and “views” or “analysis and recommendations” are available. Expert meetings before 7 April 2020 are listed in Table 4.

### 4.1. Evolution of Testing Strategy and Contact Tracing

On 7 February 2020, the first advisory board meeting (Meeting Pre-1) was convened, and the infectivity of asymptomatic cases as well as the identity of the testing targets (i.e., whether asymptomatic cases could be included) were addressed [23]. In Meeting Pre-2, the significance of expanding the PCR testing capacity by using academic laboratories was critically discussed. How to determine the virus load in asymptomatic cases was also a topic of debate [24]. In Meeting 1, the necessity of increasing surveillance sensitivity and clarifying testing targets and testing purpose was discussed [25]. By reflecting on lessons learned from the 2009 H1N1 influenza pandemic, the discussion was centered on how to restrain the number of visits to outpatient clinics for preventing nosocomial infections in Meeting 2 [26]. At this moment, the testing capacity was already on the verge of being overwhelmed in some regions. Whilst the importance of early diagnosis was noted in Meeting 6, “early detection and response to clusters” were simultaneously emphasized [30,37,38], demonstrating reactive responses to emerging clusters. Telemedicine and constrained access to testing were also discussed in Meeting 8 [32,40,41]. There was a clear argument that “lack of testing delayed the detection of nosocomial infections” in Meeting 10 [34]. It was concretely noted that there were many suspected COVID-19 cases whose testing requests from front-line healthcare workers were rejected by public health centers [34].

### 4.2. Issues in Health System Capacity

A mismatch between the primary objectives of beds for infectious disease patients in designated medical institutions and the severity of admitted patients was noted in Meeting 1 [25]. The highest priority of “effective number of hospital beds” that contemplates human resources was mentioned in Meeting 4 [28]. Additionally, a critical voice on the front line, which argued that the increase of severe cases oppressed the capacity of intensive care units (ICU) and depleted personal protective equipment (PPE) increased the risk of nosocomial infections, was delivered to the expert meeting [28]. Estimated pandemic planning scenarios that instructed local governments to increase health system capacity were approved in Meeting 5 [29], and “enhancement of intensive care and securing of a medical service system for the severely ill” were listed as one of three basic strategies in Meeting 6 [30,37,38]. Tragic scenarios were presented in Meeting 8, which implied that the number of severe cases would outweigh the number of ventilators available [40]. The uneven burden in designated medical institutions for infectious diseases was repeatedly discussed. In Meeting 10, the sluggish preparedness for expanding health system capacity was condemned, and the re-allocation of mild cases, exhaustion of PPEs, and logistics for testing—especially, how to secure the healthcare workforce—were critically debated [34,42,43].

### 4.3. Border Control

In the Pre-1 meeting, the necessity of a stepwise review of border control was agreed upon [23]. However, border control according to a science-based risk assessment was not discussed for over a month. In Meeting 6, the expansion of the epidemic in foreign countries was addressed [30,37,38], and this became a critical issue in Meeting 7 on 17 March 2020, when members raised serious concerns for an increasing number of imported cases and urged the government to test and isolate all passengers from designated areas of immigration restrictions and to impose a 14-day quarantine to returnees and visitors from non-designated countries [39].

### 4.4. Health Communication

In the Pre-2 Meeting, members asked the government to present future scenarios for gradually strengthening domestic containment, as more attention had been paid to the Diamond Princess cruise ship [24]. In Meeting 1, members argued that younger adults played a critical role in driving the infection and asked the government to present its understanding on the severity of the virus. It was also communicated that the infection had already spread to some regions, and insufficient capacity in testing, contact tracing, and isolation would be a bottleneck for promptly containing COVID-19 clusters [25]. In Meeting 3, members argued the necessity of local lockdown when chains of clusters were detected. Also, the expert meeting again urged the government to communicate the national consensus on the severity of COVID-19, as there had been some cases of “long COVID” reported among younger generations [27].

In Meeting 6, three environmental conditions increasing the risk of COVID-19 transmission were presented: “closed space with poor ventilation”, “crowded space with many people”, and “conversations and vocalization in close proximity (within arm’s reach of one another)” [30,37,38]. “Behavior modification of citizens” was noted as one of three pillars of Japan’s strategy, but the minutes concurrently elucidated a discussion on the fact that the anticipated long-term countermeasures must be effectively communicated to the public, otherwise regional lockdown, at the very least, would become inevitable [30]. Also, it was asserted that decreasing social contacts in specific periods and clarifying public health communications, such as the importance of physical distancing, by illustrating evidence in China, should be the center of attention [30]. Simultaneously, there was an argument that the effectiveness of ventilation, sterilization, and wearing masks must be communicated without downgrading the importance of decreasing social contacts [30].

Tension among members of the expert meeting appeared after Meeting 8 [32]. While members argued that combating COVID-19 would be a long-term continuous battle and discussed the limitations of the ongoing strategy and the necessity of revising it, some emotional objections were raised [32]. At this moment, discrimination against healthcare institutions and healthcare workers was firstly discussed. Despite the surge of COVID-19 cases, the necessity of behavior modification campaigns for avoiding the “3Cs: closed spaces with poor ventilation, crowded places with many people nearby, and close contact settings such as close-range conversations” was peculiarly discussed again in Meeting 10, and members did not urge the government to impose draconian measures in Meeting 10 [34,42,43].

## 5. Discussion

### 5.1. Obscure Decision-Making Process of Testing Strategy and Effective Retrospective Contact Tracing

This study clarified that the importance of expanding the diagnostic capacity by using university and large private laboratories and the necessity of capturing asymptomatic infections were the main topics in the early phase of the debate [23,24,25]. This corresponded to the most important issues at that point, which regarded Japan’s testing capacity [19], the identification of asymptomatic infections [76,77,78,79,80,81,82], estimates of the magnitude of COVID-19 [83,84], and challenges in infection control [85].

Conversely, these accumulated pieces of evidence were not fully utilized to organize a proper COVID-19 response in Japan. Whereas testing all suspected cases is crucial for the pandemic response [86], only a small proportion of infected cases was captured with Japan’s testing strategy. This tactic could be defended on the basis of the previous response to the H1N1 pandemic in Kobe in 2009 [87], but it was also claimed that tailor-made approaches depending on public health resources in local areas were essential to prevent citizens from rushing to hospitals [88]. Considering Japan’s experienced of a pre-symptomatic infection during the H1N1 pandemic [89] and the accumulated epidemiological data of asymptomatic infections of COVID-19 [76,77,78,79,80,81,82], the choice of restrictive access to testing as a valid measure could be debatable. As argued in expert meetings, insufficient testing caused many nosocomial and community-acquired infections [34], and incomplete reporting as well as delays in case confirmation made it challenging to capture the magnitude of the epidemic [12,90,91,92,93,94]. Adopting drive-through testing, which became popular in South Korea, was not seriously considered. While the safety measures were relaxed in early May [95], the decision-making process of restrictive standards of testing consultation and the real efforts made both at the national and at local levels to ensure citizens’ rights to healthcare, including testing, must be verified.

Retrospective contact tracing, while pre-modern and manually conducted, effectively worked for exploring sources of infection in the early phase of the epidemic and captured the evolution of transmission dynamics. Overdispersion of the reproduction number, which was argued theoretically in mid-February [96], was presented from real-world data, and environmental risks factors that might cause superspreading events were denoted [97]. However, the objectives and limitations of a cluster-based approach were not clearly presented. In a strange turn of events, the expert committee stuck to this tactic even after the surge of COVID-19 cases in early April. While there were positive judgments of this tactic [98,99], its efficiency in delaying the surge of the infection has not been quantitatively reviewed. Furthermore, it is doubtful whether government officials truly acknowledged the significance of early interventions to interrupt viral transmission. These could be understood by divulging transcripts of expert meetings.

### 5.2. Issues in Health System Capacity

There was a sluggish response to expanding health system capacity by local governments. In principle, patients with designated infectious diseases, specified under the Infectious Diseases Control Law, had to be admitted to designated institutions. Though the Ministry of Health, Labor, and Welfare stated that mild cases had not to be necessarily admitted to hospitals, which was presumably based on discussion in expert meetings [28,29,36], these decisions were not communicated persuasively. Therefore, even asymptomatic infections were admitted to hospitals, placing additional significant burden on these institutions. The overstretched capacity of designated hospitals and the lack of a coordination system between stakeholders, including non-designated hospitals and local governments, were recognized as challenges.

Also, the Achilles’ heel of Japan’s health system must be noted. The number of hospital beds per population was much higher than the average in countries of the Organization for Economic Co-operation and Development (OECD) [100]; however, the number of ICU beds was limited to 7.3 per 100,000 population, which was lower than in other Asian high-income countries and some European high-income countries [101,102]. In addition to the relatively lower number of doctors per population [100], the insufficient number of experts in infectious diseases and intensive care became a critical matter. Furthermore, even before the COVID-19 pandemic, the wide-ranging engagement of Japanese physicians was reported [103], and the pandemic aggravated the health conditions of healthcare workers [104]. Telemedicine had not been broadly introduced before the COVID-19 pandemic. Nevertheless, issues concerning the health workforce were insufficiently addressed, in contrast to the numbers of beds and ventilators, which were repeatedly discussed.

While efforts by front-line healthcare workers could mitigate the impact of COVID-19, Japan faced many unprecedented challenges. Japan’s healthcare system was constrained and nearly collapsed, especially in metropolitan areas. In Tokyo, in April 2020, the number of rejections in emergency transport nearly quadrupled compared to April 2019 [105]. Both Japanese Association for Acute Medicine and Japanese Society for Emergency Medicine declared in early April that they recognized the collapse of the emergency medical care system [106]. The number of postponed elective surgeries amounted to more than 100,000, which was much larger than in other countries [107]; the rate of childhood routine immunizations was lower [108]. Finally, no publicly available data have been presented on how many healthcare workers contracted COVID-19 and died from it. While a sector-wide approach is crucial to maintaining these essential health services, these were not discussed enough, and challenges are still unsolved.

### 5.3. Sluggish Reaction for Border Control and Lack of Harmonization

A sluggish governmental response towards COVID-19 in border control was evident, and it is doubtful whether a science-based risk assessment was conducted. When strict control measures were imposed in Wuhan, resulting in a complete city lockdown on 23 January 2020 [109], Japan did not impose any border control measures. The launch of COVID-19 response headquarters and the first implementation of border control was done after the Chinese festival holiday period [45].

Moreover, when COVID-19 cases exploded in European countries in March, the governmental response became much slower. Our study suggests that before mid-March, the expert meeting did not significantly address issues regarding border control and quarantine. Simultaneously, there might have been the politicization of quarantine and immigration policies in this period. While the expert meeting urgently asked the government to impose strict measures on 17 March 2020 [31], the government strengthened the quarantine on 21 March 2020. Considering the media report suggesting that the arrival of the Olympic torch on 20 March 2020 delayed the decision-making in border control [110], whether the government truly imposed a border control on the basis of a science-based risk assessment or prioritized political needs should be examined by clarifying the decision-making process.

Finally, before and during the state of emergency, even symptomatic patients suffered from interrupted access to testing [12]. On the contrary, all passengers from designated countries and areas, regardless of being symptomatic or asymptomatic, were screened by PCR test [56,57]. Considering that insufficient testing became a critical issue domestically and that a large amount of testing was performed on refugees, optimal resource allocation should have been seriously considered in conjunction with strengthening border control, for example by combining 14-day isolation in specific facilities with testing in an appropriate timeframe depending on refugees’ departure places and dates. To tackle these challenges, reviewing the current unconstructive sectionalism and building coordinated structures with command and control will be vital [12].

### 5.4. Challenges in Health Communication

Japan’s weakness in health communication, which was acknowledged even before the COVID-19 pandemic, has become evident [12,111]. Commonly, risk communication is a task of the press secretary or other government officials. However, members of the expert meeting started to hold a press conference from late February onwards to complement the Ministry’s role. The press conference played a critical role in raising awareness and preparedness among media and citizens by ensuring openness and transparency regarding the epidemiological risk assessment. For example, the importance of decreasing social contacts was repeatedly communicated from the earlier phases [30,55] and helped a risk-informed decision-making. This measure was different from those of other countries, such as an upper limit of participants for social gatherings and events issued by the Centers for Disease Control and Prevention [112], and might allow citizens to understand the underlying transmission patterns of COVID-19. Nevertheless, members in the expert meeting acted as if they were all responsible for the decision-making of the governmental policy.

This study also clarifies that there was misunderstanding in differentiating risk communication and crisis communication between the expert committee and governmental officials [12]. While there was abundant risk communication in early March, such as messages for younger adults and emphasis on avoiding the “3Cs” [36,37,38,40,41,42,43], there was no transition to crisis communication, which constitutes the core of communication strategies in the response to health emergencies as it delivers direct, clear, and concise messages [113,114,115,116,117]. Even when the expert meeting acknowledged that COVID-19 was prevalent in Japan in mid-March [32] and Japan was under a surge of COVID-19 in early April [34,42,43], risk communication stressing the importance of avoiding the “3Cs” was continued. As both risk communication (i.e., avoiding the “3Cs”) and crisis communication (i.e., physical distancing, washing hands frequently, staying home, and protecting health systems) were delivered to the public in parallel without strengthening specified messages, crisis communication was not effective. As a matter of fact, an online survey conducted on 31 March–1 April 2020 clarified that only 32.8% of the citizens avoided conversations in close-contact settings [118]. This explicitly suggested that the importance of physical distancing was not persuasively communicated in February–March. Also, as the interpretation of the “3Cs” was different among citizens and difficult especially for vulnerable people, only less than 30% of the citizens had modified their behavior in early April [119]. Nevertheless, the primary focus on the “3Cs” continued even under the state of emergency in April–May, demonstrating the government’s inability to differentiate risk communication from crisis communication. A lack of experts in media and communication during the expert meetings might be responsible for this flaw [12].

Additionally, it is necessary to examine why the expert meeting continued to emphasize behavior modification campaigns and avert from strict measures, as the effectiveness of the lockdown in reducing the spread of the infection number has been scientifically demonstrated [109,120]. Conversely, scant evidence supports the effectiveness of behavior modification campaigns in the early phase of the pandemic [12]. The emphasis on the behavior modification campaigns is in contradiction to the logic utilized for deciding restricted access to testing [26,87,88]. Finally, the effects of the strategy on the spread of COVID-19 and on future trends of epidemic evolution were not communicated by governmental officials. The tension between politics and science in Japan has already been pointed out [12], and whether “views” or “analysis and recommendations” presented by the expert meeting were truly based on scientific evaluation or distorted by the government needs to be examined.

The strategy to ensure delivering timely, clear, concise, and informative communications to the public is pivotal to sustain the faith of citizens towards the government, deepen solidarity, and protect vulnerable populations, regardless of their nationality, age, and gender. Maintaining public health communication with the public is indispensable, and investment in health communication, such as establishing the position of health communication officers or allocating competent political executives to manage public-health decisions must be earnestly considered.

### 5.5. Early Actions by Local Governments but Insufficient Community Engagement and COVID-19-Related Social Issues

In opposition to the discrepancy between the latest risk assessment and governmental intentions, which is explicitly illustrated by the request of nationwide school closure in late February, and multiple challenges in health communication as argued before, early and decisive actions at local levels compensated for poor leadership at the national level. In Wakayama, aggressive testing, contact tracing, and isolation made it possible to promptly contain the spread of COVID-19, which was praised as the “Wakayama model” [121]. The determination of requesting school closure and declaring a local state of emergency by Hokkaido governor in late February helped decrease the infection spread in March [122]. Suggestions for imposing a lockdown in Tokyo and repeated warnings through a press conference by Tokyo governor in late March contributed to raise awareness among citizens [54]. In fact, the effective number of COVID-19 cases significantly decreased to less than one in late March [123], demonstrating a strong commitment of the citizens to comply with these warnings, although these lacked in legal enforceability, and a financial compensation was not ensured.

Concomitantly, downsides of these voluntary schemes must be acknowledged, as many social issues related to COVID-19, including anxiety and fear among citizens, as well as discrimination and social sanctions to Chinese visitors, COVID-19 patients, their families, and healthcare workers on the front line emerged in Japan [111,124]. Sensational media reports also spurred on creating a kind of surveillance society and stigmatization [111,125], and inadequate community engagement, which is critically recognized in any outbreak [126], was recognized in Japan. As criticism toward COVID-19 patients generated hesitancy to testing among citizens, it has become more difficult to capture the transmission dynamics.

### 5.6. Limitations

Several limitations should be noted. First, while our research could review the domestic response to COVID-19 in Japan, this could not sufficiently address issues in unprecedented quarantines. This is because the expert meeting was launched in mid-February, and its main focus was on how to tackle the domestic spread of COVID-19. Second, while we discussed Japan’s response to COVID-19 from several perspectives as presented in previous literature [12], this conceptualization could not fully cover whole dimensions in medicine and public health. For example, due to the nature of the expert meeting, issues in health financing were not addressed enough. Therefore, extracting actual materials published by public institutions and employing a theoretical framework on core dimensions of health system resilience during outbreaks will help close the gap between actual needs on the front line and governmental action. Third, general challenges of the content analysis approach, such as inherent reductionism or the difficulty of replication, should be noted [127]. These could be compensated by reviewing the quantitative analysis and conducting a qualitative interview. Despite these limitations, this research presents both beneficial and critical features of Japan’s COVID-19 response in the early phase of the epidemic. As suggested in other countries, launching an independent panel for conducting an external evaluation will help improve a country’s COVID-19 response [17,128,129]; our research results support this conclusion and will be referenced in such circumstance.

## 6. Conclusions

Japan has confronted many challenges regarding epidemiology, health system capacity, border control, and health communication during the first wave of COVID-19. Experience in managing quarantines and rapid, scientific risk assessment in the early phase of the epidemic were not fully utilized due to logistical issues and a lack of early and decisive governmental action at the national level. Some interventions at several levels were not harmonized enough, and health communication was not efficient, which resulted in social issues related to COVID-19. It is true that Japan managed to avoid an explosive surge of COVID-19 cases in April, but Japan needs to face the epidemiological and economic impacts of COVID-19. Examining previous responses will be vital to detect the root causes of issues. Not only gathering successful responses but summarizing lessons learned from previous mistakes in each country or region will contribute to continuously improve the global response to COVID-19. Ensuring openness and transparency, derivatizing transcripts of expert meeting, and clarifying the decision-making process of each countermeasure will help tackle many challenges. Investing in public health, strengthening the capacity of rapid risk assessment, harmonizing countermeasures managed by different ministries at the national level, and reinforcing health communication will improve the COVID-19 response in Japan. Moreover, government officials must demonstrate their leadership to effectively communicate public health messages. To our knowledge, this is the first study that objectively examines Japan’s responses to the first wave of COVID-19. This paper will be utilized as a benchmark to kick off the debate on how challenges were confronted and measures decided during the first wave so to prepare for the winter season as well as the next pandemic.

## Figures and Tables

**Table 1:** Key policy measures in Japan’s response to COVID-19, January–May, 2020.

Periods	Date	Events/Policy Measures
(i)	16 January 2020	First COVID-19 patient reported
	28 January 2020	COVID-19 cases without travel history to Wuhan reported
	29 January 2020	Wuhan repatriation mission started
	30 January 2020	COVID-19 response headquarters launched
	3 February 2020	Quarantine of the Diamond Princess started in Yokohama
	17 February 2020	Consultation criteria for COVID-19 testing published by the Ministry of Health, Labor, and Welfare
(ii)	25 February 2020	“Basic Policies for Novel Coronavirus Disease Control” introduced, and cluster-response section launched by the Ministry of Health, Labor, and Welfare
	26 February 2020	School closure in Hokkaido requested by Hokkaido governor
	27 February 2020	Nationwide school closure requested by PM Abe
	28 February 2020	Local state of emergency declared in Hokkaido
	19 March 2020	Local state of emergency lifted in Hokkaido
	20 March 2020	Nationwide school closure request cancelled
	23 March 2020	Lockdown in Tokyo suggested by Tokyo governor
	24 March 2020	Postponement of the Tokyo 2020 Olympics and Paralympics announced
	25 March 2020	Explosive increase of COVID-19 cases declared by Tokyo governor
(iii)	7 April 2020	State of emergency declared in seven prefectures
	16 April 2020	State of emergency expanded to the entire nation
	4 May 2020	State of emergency period expanded until the end of May
	14 May 2020	State of emergency lifted in 39 prefectures
	21 May 2020	State of emergency lifted in three prefectures in Kansai region
(iv)	25 May 2020	State of emergency lifted in all prefectures

**Table 2:** Border control in Japan before the state of emergency on 7 April 2020 [58,59,60,61,62,63,64,65,66].

Date	Designated Areas of Immigration Restrictions under the Immigration Control Act	Countries Subject to Strengthened Quarantine
1 February 2020	China (Hubei)	
13 February 2020	China (Zhejiang)	
27 February 2020	Partly: Republic of Korea	
7 March 2020	Partly: Republic of Korea, Iran	
9 March 2020		China, Republic of Korea
11 March 2020	Partly: Iran Entirely: Italy, San Marino	
19 March 2020	Partly: Italy, Switzerland, Spain Entirely: Iceland	
21 March 2020		Schengen countries (Iceland, Italy, Estonia, Austria, Netherland, Greece, Switzerland, Sweden, Spain, Slovakia, Slovenia, Czech Republic, Denmark, Germany, Norway, Hungary, Finland, France, Belgium, Poland, Portugal, Malta, Latvia, Lithuania, Liechtenstein, Luxembourg), Ireland, Andorra, Iran, United Kingdom, Egypt, Cyprus, Croatia, San Marino, Vatican City, Bulgaria, Monaco and Romania
26 March 2020		United States of America
27 March 2020	Ireland, Andorra, Italy, Estonia, Austria, Netherland, Switzerland, Sweden, Spain, Slovenia, Denmark, Germany, Norway, Vatican City, France, Belgium, Portugal, Malta, Monaco, Liechtenstein, Luxembourg, Iran	
28 March 2020		Israel, Qatar, Bahrain, Democratic Republic of the Congo, Indonesia, Singapore, Thailand, Philippines, Brunei, Vietnam, and Malaysia
3 April 2020	Albania, Armenia, Israel, Indonesia, United Kingdom, Ecuador, Egypt, Australia, Canada, South Korea, Northern Macedonia, Cyprus, Greece, Croatia, Kosovo, Democratic Republic of Congo, Ivory Coast, Singapore, Slovakia, Serbia, Thailand, Taiwan, Czech Republic, China (including Hong Kong and Macau), Chile, Commonwealth of Dominica, Turkey, New Zealand, Panama, Hungary, Bahrain, Philippines, Finland, Brazil, Bulgaria, Brunei, United States of America, Vietnam, Bosnia and Herzegovina, Bolivia, Poland, Malaysia, Moldova, Morocco, Montenegro, Mauritius, Latvia, Lithuania, Rumania	All countries and regions

**Table 3:** COVID-19 cases and deaths in Japan by age group, as of 27 May 2020 (6 p.m.) [75].

Age Group	Confirmed Cases	Deaths	Case Fatality Risk (%)
80+	1783	327	18.3%
70–79	1637	160	9.77%
60–69	1865	66	3.54%
50–59	2733	20	0.73%
40–49	2620	9	0.34%
30–39	2502	4	<0.01%
20–29	2717	0	0%
10–19	390	0	0%
0–9	278	0	0%
Total	16,575	586	3.53%

Notes: As 50 cases were unknown, under investigation, or unpublished as regards their age group, the number of total confirmed cases does not correspond to the sum of the data in each age group. Source: The Ministry of Health, Labor, and Welfare, Japan (Publicly available data. No copyright issue).

**Table 4:** Novel coronavirus expert meetings in Japan, before 7 April 2020.

Date	Meeting Number *	Minutes	Views/Analysis and Recommendations
7 February 2020	Pre-1	Yes [23]	No
10 February 2020	Pre-2	Yes [24]	No
16 February 2020	1	Yes [25]	No
19 February 2020	2	Yes [26]	No
24 February 2020	3	Yes [27]	Yes (JPN) [35]
29 February 2020	4	Yes [28]	No
2 March 2020	5	Yes [29]	Yes (JPN) [36]
9 March 2020	6	Yes [30]	Yes (JPN/ENG) [37,38]
17 March 2020	7	Yes [31]	Yes (JPN) [39]
19 March 2020	8	Yes [32]	Yes (JPN/ENG) [40,41]
26 March 2020	9	Yes [33]	No
1 April 2020	10	Yes [34]	Yes (JPN/ENG) [42,43]

* As the first two meetings were held as advisory board meetings at the Ministry of Health, Labor, and Welfare and their nature was slightly different from expert meetings in governance, they are indicated by “pre-numbers”. Abbreviation: JPN, Japanese; ENG, English.
